# The prion dilemma confounding science educators

**DOI:** 10.12688/f1000research.2-4.v1

**Published:** 2013-01-09

**Authors:** Igor V Zaitsev, Ling Chen, Susie Boydston-White, Manita Pavel, Svetlana N Shugaeva, Alla G Petrova, Oksana V Williams

**Affiliations:** 1 Borough of Manhattan Community College, The City University of New York, New York, 10007-1097, USA; 2 Irkutsk State Medical Academy of Postgraduate Education, Irkutsk, 664003, Russian Federation; 3 Irkutsk Medical University, Irkutsk, 664003, Russian Federation; 4 American International School of Bucharest, Bucharest, 077190, Romania

## Abstract

In this paper, the issue of the prion hypothesis, a simmering controversy within the scientific community, is addressed. We inquire into the appropriateness of the use of certain augmentations and rhetoric approaches used during scientific debates, as well as the aptness of unequivocal statements in textbooks that indicate “abnormal prions” as a primary cause of Transmissible Spongiform Encephalopathies.

## Correspondence

According to some in the field, one should refrain from discussions concerning controversial issues in science if one is not actively conducting experimental research ^1^. We must dissent, most particularly when the prions controversy is under consideration. One does not have to conduct scientific experiments to recognize not only the flaws of the prion protein (PrP) hypothesis ^2^, but the inappropriate vocabulary used during discussions of the issue. As science educators, we are still confounded when trying to present the cause of Transmissible Spongiform Encephalopathy (TSE) to our students.

To start with, for the past twenty years, the majority of biology text books unequivocally identified PrP ^Sc^ as the causative agent of TSE, and some texts even refer to the “prion hypothesis” as the “prion theory”, please see Table 1. Yet, when introducing the scientific method in high schools and college classes, we establish that in order for a hypothesis to become a scientific theory, it has to be supported many times over through experimentation ^3^ providing a substantial and conclusive body of evidence ^4^. Upon reviewing experimental work on PrP, one notes that initial studies are rarely, if ever, repeated by other scientists. Instead, they move on without giving reconsideration to the assumption upon which they base their work ^5^.

**Table 1.** The indisputable textbook statements concerning infectious agent of Transmissible Spongiform Encephalopathies.

Authors	Name of the textbook	Publishing company	Year of the publication	Statements
McKee T., McKee J.R.	Biochemistry: The molecular Basis of Life	McCraw Hill	2003	“Prion disease are caused when the conformation of PrP ^C^ is converted to PrP ^Sc^”.
Gladwin M., Trattler W.	Clinical Microbiology Made Ridiculously Simple	MedMaster	2004	“The prion-only hypothesis is the most widely accepted theory today”.
Freeman S.	Biological Science	Pearson Benjamin Cummins	2008	“Over the past several decades, evidence has accumulated that certain proteins can act as infectious, disease causing agents”.
Russell P.J., Wolfe S.L., Hertz P.E., Starr C., McMillan B.	Biology: the Dynamic Science	Thomson Brooks/Cole	2008	“Prions … are the only known infectious agents that do not include a nucleic acid molecule”. “Prions have been identified as the causal agents of certain diseases that degenerate the nervous system in mammals”.
Campbell M.K., Farrell S.O.	Biochemistry	Thomson Brooks/Cole	2009	“It has been established that the causative agent of mad-cow disease, as well as the related diseases scrapie in sheep, chronic wasting (CWD) in deer and elk, and human spongiform encephalopathy in humans is a small (28-kDa) protein called a prion”.
Tymoczko J.L., Berg J.M., Lubert S.	Biochemistry: A Short Course	W.H. Freeman & Company	2010	“Certain infectious neurological diseases were found to be transmitted by agents that were similar in size to viruses but consisted only of protein”.
Talaro K.P.	Foundations in Microbiology	McGrow Hill	2009	“Prions are incredibly hardy “pathogens”. They are known to cause diseases called transmissible spongiform encephalopathies”.
Cowan M.K., Bunn J.	Microbiology Fundamentals: A Clinical Approach	McGrow Hill	2013	“The transmissible agent in CDJ is a prion”.
Tortora G.J., Funke B.R., Case C.L.	Microbiology: An Introduction	Pearson	2013	“Several fatal diseases affecting the human central nervous system are caused by prions”.

When describing the scientific method, it is important that we emphasize the difference between faith and fact. Nevertheless, during discussions of the PrP hypothesis in meetings, conferences and private discussions of scientists, “I think” is too often replaced by “I believe”. Perhaps, this inclination began when the Karolinka neurologist Lars Edison told *The Times* newspaper upon the announcement of the Prusiner’s Noble Prize: “There are still people who don’t believe that a protein can cause these diseases, but we believe it” ^6^. There should be no place in science for such a subjective declaration. Even recent publications emphasize that the scientific community has been split into PrP “believers” and “nonbelievers”. Laura Manuelidis, one of the main scientists who rejects the PrP hypothesis, has been portrayed as a “prion heretic” ^7^. Upon entering the combination of “prions” and “belief” in a Google search, we generated an astonishing 918,000 hits. Another recent tendency in modern science is marginalizing scientists as the “minority” versus the “majority” as is seen in the PrP controversy ^7^, a partition more suitable for political rather than scientific discussions.

In covering the PrP hypothesis in classrooms, are we also to employ a vocabulary in which the scientific community is divided into “believers” and “nonbelievers” or “majority” and “minority” as if we were referring to a religious conviction or a political debate rather than a scientific dilemma?
