# Supplementary material for: The prion dilemma confounding science educators
Source: F1000Res. 2013 Jan 9;2:4. [Version 1] doi: 10.12688/f1000research.2-4.v1 (PMC3894800; doi:10.12688/f1000research.2-4.v1)
Supplement: Peer review history

# Peer review: Referee response for version 1

**Published:** 2013-09-09

**Authors:** Hidehiro Mizusawa

**Affiliations:** 1 Department of Neurology and Neurological Science, Tokyo Medical and Dental University, Tokyo, Japan

I agree with the authors on how important open discussion is in science. However, the prion hypothesis has been well and openly discussed for many years. Due to the hypothesis, many achievements have been obtained. Abnormal prion proteins resulting from prion protein gene mutations clearly cause genetic prion diseases.

Minor point: “Abnormal prions” should be “prions”, because prions all are abnormal.

I have read this submission. I believe that I have an appropriate level of expertise to confirm that it is of an acceptable scientific standard.

# Peer review: Referee response for version 1

**Published:** 2013-01-25

**Authors:** Kai Zinn

**Affiliations:** 1 Division of Biology, California Institute of Technology, Pasadena, CA, USA

The scientific community has been split in the past into those who believed the prion hypothesis and those who did not.  During the 1980s and part of the 1990s, most work on the prion hypothesis was from Stanley Prusiner's group, and those who questioned the prion hypothesis were doing so largely by finding potential errors in the work of one laboratory.  However, now we have hundreds of papers on mammalian PrP, including, most importantly, the demonstration that transmissible disease can be caused by a pure recombinant prion protein ( Wang *et al*, (2010)), that are not from Prusiner and whose results are consistent with the prion hypothesis.  In addition, work by many groups on yeast prions demonstrated the validity of the generalized prion hypothesis (inheritance mediated by conformational changes in proteins) in a more experimentally tractable system in which controls that were not possible for mammalian PrP could easily be done. So, at this point, I see no problems with the statements made in the textbooks that are listed in the Table. The prion hypothesis is as well-established, at least for mammalian PrP, as the chemiosmotic (Mitchell) hypothesis for ATP synthesis by mitochondria, which was controversial at the time it was proposed in the early 60s, but which is now the only mechanism described in textbooks. It is no longer necessary to even mention the alternative ideas from the 60s, such as chemical coupling.

I have read this submission. I believe that I have an appropriate level of expertise to confirm that it is of an acceptable scientific standard, however I have significant reservations, as outlined above.

# Peer review: Referee response for version 1

**Published:** 2013-01-17

**Authors:** Jose Valpuesta

**Affiliations:** 1 Centro Nacional de Biotecnologia, Campus Universidad Autónoma de Madrid, Madrid, Spain

I agree with what I think is the main message of the authors, that the scientific debate should be open and should rest in facts and not in beliefs. The first point is very important and the same 'prion hypothesis' is a good example of this, as it was under attack for a long time until substantial evidence was produced in its favour. I agree with the authors in that in science one should refrain from using statements ('I believe ...') more adequate for religious or political debates, but when dealing with educational matters, simple statements need be used to convey a certain message or information. Time will tell whether these messages are correct, and the text books and scientific journals are full of information that later has been proven to be wrong, but which has been useful to stir the scientific debate.

I have read this submission. I believe that I have an appropriate level of expertise to confirm that it is of an acceptable scientific standard.
